# Pleasantly Flavored

**Published:** 1882-01

**Authors:** 


### PLEASANTLY FLAVORED.

EVERY Frenchman's food is flavored before it comes to table, and it is artistically and wholesomely done. Where we deluge our food with highly-spiced sauces and hot pickle-juice, in order to "give it a taste," the French use delicate vegetable essences and savory herbs. Carrots, turnips, onions, leeks, cabbage, mushrooms and truffles, together with tarragon, thyme, chervil, laurel and parsley, form the basis of French flavors. Spices they use in exceeding moderation. The essence of common vegetables and pot-herbs, combined with the juices of various meats, is the basis of all their sauces. The almost infinite variety of combinations which can be made of these simple ingredients is astonishing. This great variety of flavors keeps the palate constantly amused, as it were, whereas the eternal monotony of the average English kitchen results in a sort of gastronomic boredom, which is to be combated only by such violent means as strong mustard, cayenne, and that esculent fire known as "hot pickles." So perfectly is the food flavored by French cooks that at table not even salt need be added to it.

